# Differences in Sexual Practices, Sexual Behavior and HIV Risk Profile between Adolescents and Young Persons in Rural and Urban Nigeria

**DOI:** 10.1371/journal.pone.0129106

**Published:** 2015-07-14

**Authors:** Morenike Oluwatoyin Folayan, Sylvia Adebajo, Adedayo Adeyemi, Kayode Micheal Ogungbemi

**Affiliations:** 1 Institute of Public Health and Department of Child Dental Health, Obafemi Awolowo University, Ile-Ife, Nigeria; 2 Population Council, 16, Mafemi Crescent, Abuja, Nigeria; 3 MEASURE, 90 Nelson Mandela Street, Asokoro, Abuja, Nigeria; 4 Strategic Knowledge Management Unit, National Agency for the Control of AIDS, Abuja, Nigeria; University of Georgia, UNITED STATES

## Abstract

**Introduction:**

We aimed to determine differences in sexual practices, HIV sexual risk behaviors, and HIV risk profile of adolescents and young persons’ in rural and urban Nigeria.

**Methodology:**

We recruited 772 participants 15 to 24 years old from urban and rural townships in Nigeria through a household survey. Information on participants’ socio-demographic profile (age sex, residential area, number of meals taken per day), sexual practices (vagina, oral and anal sex; heterosexual and homosexual sex; sex with spouse, casual acquaintances, boy/girlfriend and commercial sex workers), sexual behavior (age of sexual debut, use of condom, multiple sex partners, transactional sex and age of sexual partner), and other HIV risk factors (use of alcohol and psychoactive substances, reason for sexual debut, knowledge of HIV prevention and HIV transmission, report of STI symptoms) were collected through an interviewer administered questionnaire. Differences in sexual behavior and sexual practices of adolescents and HIV risk profile of adolescents and young persons resident in urban and rural areas were determined.

**Results:**

More than half (53.5%) of the respondents were sexually active, with more residing in the rural than urban areas (64.9% vs 44.1%; p<0.001) and more resident in the rural area reporting having more than one sexual partner (29.5% vs 20.4%; p = 0.04). Also, 97.3% of sexually active respondents reported having vaginal sex, 8.7% reported oral sex and 1.9% reported anal sex. More male than female respondents in the urban area used condoms during the last vaginal sexual intercourse (69.1% vs 51.9%; p = 0.02), and reported sex with casual partners (7.0% vs 15.3%; p = 0.007). More female than male respondents residing in the rural area engaged in transactional sex (1.0% vs 6.7%; p = 0.005). More females than males in both rural (3.6% vs 10.2%; p = 0.04) and urban (4.7% vs 26.6%; p<0.001) areas self-reported a history of discharge. More females than males in both rural (1.4% vs 17.0%; p = 0.04) and urban (15.0% vs 29.1%; p<0.001) areas self-reported a history of itching.

**Conclusion:**

There are differences in the sexual behavior and practices of adolescents and young persons’ residing in the urban and rural area with implication for HIV prevention programming.

## Introduction

Nigeria is home to the second largest HIV and AIDS epidemic in the world ^1^. An estimated 3.4% (3,229,757 people) of the population were living with HIV [1] by the end of 2013 [1]. The rate of new infections has started to decline: (283,589 in 2010, to 220,394 in 2013) [1,2], but the total number of new infections in females continues to surpass that of the males [1,2]. The estimated number of new infections in adolescents and young persons’ age 15 to 24years also show similar trends: the number of new HIV infections dropped from 74,783 in 2009 to 54,662 in 2013. The number of new infections in female adolescent and young persons’ surpasses that in males [1].

The main mode of transmission of HIV in Nigeria is sexual with 42% of new infections occurring amongst persons practicing ‘low-risk’ sex – married and co-habiting sex partners [3]. High risk sexual behavior and practices for HIV transmission include early sexual debut, inconsistent use of condom during sex, having multiple sex partners, having a sex partner who is older by 10 years or more, engaging in transactional sex, and having unprotected anal sex [4]. A recent secondary data analysis assessed changes in high risk sexual behavior of adolescents in Nigeria aged 15–19 years over a five years period (2007 and 2013) showed that there was significant decrease in the proportion of female adolescents who became sexually active before the age of 15 years; and the use of condom at last sexual act with non-marital sexual partners had significantly increased [4]. These finding were consistent with global reports on sexual behavior of adolescents and young persons [5].

A field study conducted among a representative sample of adolescents 10–19 years in Nigeria showed significant differences in male and female adolescents’ high risk sexual behavior. Significantly more females than males engaged in transactional sex, had sex with partners who were 10 years or more older than them, and practiced oral sex. Being a female, and having engaged in anal sex in the last 12 months reduced the odds of using a condom at last vaginal intercourse [6]. The findings of this study suggest that although fewer adolescents are engaging in sexual practices that increase their risk for HIV infection [5], priority attention needs to be given to female adolescents as they have heightened sexual risk. This finding has implications for gender mainstreaming into HIV prevention programming for adolescents in Nigeria.

Urban and rural differences have also been observed in sexual behaviors and sexual practices. For example, the urban poor are significantly more likely than their rural counterparts to have early sexual debut and a greater incidence of multiple sexual partnerships [7]. Urban women were also more likely to report condom use and multiple sexual partners [8]. Little is understood about the risk of young people who live in urban and rural areas for HIV infection and how affluent lifestyles, education and marital status of these youngsters influence their HIV risk.

There are global concerns about the HIV risk of adolescents and young persons aged 15 to 24years for contracting HIV infection. In 2010, 42% of new HIV infections in people older than 15years occurred in adolescent and young persons [6]. It is important to design evidence based programs for adolescents and young people to promote responsible sexual behavior, including the use of condoms during sexual intercourse among others [9]. This study is an effort at understanding the sexual behaviors and practices of adolescents and youths in Nigeria. It specifically examines the differences in sexual behavior (condom use with non-marital partners, multiple sexual partnerships and transactional sex), sexual practices (oral, anal and vaginal sex), HIV risk profile (knowledge about HIV transmission and prevention, use of psychoactive substances, history of sexually transmitted infection) and reasons for sexual debut among adolescents and young persons’ age 15 to 24 years living in urban and rural Nigeria.

## Methodology

We conducted a cross-sectional study with a household survey. Participants were recruited from urban and rural townships in Nigeria. Recruitment was done at National Population Enumeration sites used for 2007 National HIV/AIDS Reproductive Health Survey (NARHS) [10] and the 2010 National Antenatal Sero-sentinel (ANC) Surveys [11]Past enumeration sites used for multiple household studies on sexual and reproductive health were used based on the assumption that participants may be familiar with the conduct of such surveys and thus may likely be more open to discussions about sex.

The interviewer administered questionnaire was written in English, the national language. Some key words/phrases (including sensitive ones) for each selected community were translated into local dialects during the training of interviewers. Interviewers used the semi-translated ones as master copies. A similar approach was successfully used for the 2005 [12] and 2007^10^ NARHS, as well as the 2008 [13] and 2010 [14] Integrated Biological and Behavioral Surveillance Surveys (IBBSS) conducted in Nigeria.

### Sampling procedure

The study participants were recruited from two states – one state with low HIV prevalence and the other state with high HIV prevalence based on the 2010 ANC Surveillance Report for Nigeria [11]. States selected were Benue State (HIV prevalence of 12.7%) and Ekiti State (HIV prevalence of 1.4%).

We used a multi-stage cluster sampling procedure aimed at selecting eligible persons with known probability. Stage 1 involved selection of rural and urban localities. One urban and one rural Local Government Areas (LGA) in each of the selected states were randomly selected by balloting. The LGA listed for selection in each state were the LGA where the NARHS and the ANC surveys had been conducted in the past. Stage 2 involved the selection of enumeration areas within the selected rural and urban localities randomly selected by balloting. Stage 3 involved the listing of eligible individuals within households. At the enumeration sites, every fifth household on each street was eligible to be considered for study participants’ recruitment. Stage 4 involved selection of actual respondents for interview. Only one adolescent in each household was eligible to participate in the study. Alternative sexes were selected to participate in each consecutive household. Study participants’ recruitment continued in the enumeration site until the study sample per each LGA was reached.

### Sample size

The sample size was powered to determine the difference in anal sex prevalence between the urban and rural areas. The sample size was calculated using a prevalence of 12% of anal sex, 95% confidence interval and an error margin of 5%. The prevalence of 12% was used based on the report by Bamidele et al [15]. A minimum sample size was 162 youths age 15–24 years per LGA. This minimum sample size was increased to 194 per LGA to give room for 20% study fall out. Thus, the total sample size for the study was 776 for the four LGA.

### Data collection

A structured questionnaire was administered to respondents by experienced field workers who had been engaged in past national surveys. The field workers were trained centrally on the study protocol, the use of the data collection tools, sample selection (including household listing and selection) and all other aspects of fieldwork. In view of the complexity and sensitivity of the study, considerable amount of time was devoted to the review and role play with the questionnaire. Similar methodology had been employed with the collection of sensitive data on sex and sexuality during the NARHS and IBBSS surveys in Nigeria. The interviewers collected the information from the respondents, edited the questionnaire in the field and submitted the completed questionnaires to the survey supervisor. The supervisor reviewed all filled questionnaires and raised queries where gaps were identified in the filled questionnaire, or the consenting process. The queries were addressed latest by the next day by the field worker where this was feasible.

### Demographic and HIV risk history

The survey collected information on respondent’s socio-demographics (age at last birthday, gender, occupation, educational level and years of residence in study location), HIV risk characteristics, substance use (use of alcohol in the last four weeks and history of use psychoactive drugs), and sexual behaviors and sexual practices of respondents. The number of meals taken per day was used as proxy to assess the financial status of respondent. The number of meals per day was categorized into three: ‘one meal or less per day’, ‘two meals per day’ and ‘three meals per day’.

Participants’ knowledge of modes of HIV transmission (13 questions) and prevention (11 questions) were also assessed using questions adapted from the 2007 NARHS survey [11] The 11 questions on knowledge of mode of HIV transmission consisted of five correct responses and six incorrect responses. The 13 questions on prevention of HIV infection consisted of eight correct responses and 5 incorrect responses. Respondents were expected to give a ‘yes’ or ‘no’ response to any of the statements. Thus, the range of possible scores for knowledge on mode of transmission was 0 to 11, while the range of possible scores for knowledge on HIV prevention was 0 to13.

### Sexual behaviors and sexual practices

Participants were asked if they had ever used condoms in their lifetime, whether they had ever exchanged sex for money, a place to stay, or material goods; and the presence of STI symptoms (genital discharge, itching or sores). Participants were also asked if they had ever tested for HIV and their most recent HIV test results.

Participants were asked about the number of male and female sex partners and frequency of vaginal and anal intercourse with and without condoms in the previous three months preceding the survey. Participants were also instructed to think back over the past 90 days (3 months) preceding the survey and estimate the number of male and female sex partners they had and the number of occasions they had vaginal and anal sexual intercourse with and without condoms. These measures were similar to others that have been found reliable and valid in previous research [16].

### Data management

Respondents’ age was grouped into 15–19 years and 20–24 years. The number of years residential in study site was dichotomised: those who have lived in the study site for less than 3 years and those who had lived there for 3 years or more. Respondents’ occupation was grouped into five categories: skilled unskilled, unemployed, students, civil and servants.

Participants reporting sexual activity before age 13 years were defined as ‘early sexual debut’. Age 13 was used as the entry point for sexual initiation based on reports of an earlier study [17]. Participants reporting sexual activity before age 13 were omitted (n = 16) from the main analyses on sexual activity out of concern that they would have atypical patterns of sexual and preventive behaviors, possibly indicating abuse. Therefore, only participants with sexual debut at age of 13 or older were included in the analysis for age of sexual debut.

The scores on knowledge about HIV transmission, and HIV prevention were summed and then dichotomized into poor and good. In order to dichotomize the variables, the median of the final scores served as cut-off point, with respondents scoring below the median comprising those with poor knowledge and all others comprising those with good knowledge.

### Data analyses

The proportion of respondents with various sexual behaviors and sexual practices were analyzed. Also, the association between rural and urban residency, sexual behaviors and sexual practices were also analyzed. Pearson chi square and or ANOVA were used to test significance of associations between variables. Comparison of continuous variables was done using t-test. Statistical significance was defined at P<0.05 with a 95% confidence interval. Analysis was conducted using STATA SE version 12.0.

### Ethical Statement

The Nigeria Institute of Medical Research Institutional Review Board gave ethical clearance for the conduct of the study. Written informed consent was obtained from all study participants after duly explaining the objectives of the study, risk and benefits, voluntary nature of study participation and freedom to withdraw from the study at any time. In line with the Nigeria Labour Act [18], all National health surveys in the country had included study participants who were 15years of age. Study participants who were 15years of age and above are able to give informed consent for study participation.

## Results

### Socio-demographic profile

A total of 776 respondents were recruited for the study. There were 772 (99.5%) fully completed questionnaires used in the analysis. There were 344 (44.6%) female and 428 (55.4%) male respondents. Three hundred and fifty (45.3%) respondents reside in the rural area while 422 (54.7%) reside in the urban area (Table 1). More than half of the (56.0%) of the respondents were between ages 15 and 19 years.

**Table 1:** Socio-demographic and Sexual Health Profile of Respondents (n = 773).

Variables	Population						Total (%)
							N = 772
	Rural		p value	Urban		P value	
	N = 350						
	Male (%)	Female (%)		Male (%)	Female (%)		
	n = 201	n = 149		n = 227	n = 195		
Age							
15–19 years	100 (49.8)	84 (56.4)		138 (60.8)	110 (56.4)		432 (56.0)
20 to 24 years	101 (50.2)	65 (43.6)	0.22	89 (39.2)	85 (43.6)	0.36	340 (44.0)
Total	201 (100.0)	149 (100.0)		227 (100.0)	195 (100.0)		772 (100.0)
Occupation							
Skilled	10 (5.0)	6 (4.0)	0.68	6 (2.7)	3 (1.5)	0.52	25 (3.2)
Unskilled	48 (23.9)	17 (11.4)	0.003	5 (2.2)	10 (5.2)	0.11	80 (10.4)
Unemployed	12 (6.0)	6 (4.0)	0.42	1 (0.4)	5 (2.6)	0.10	24 (3.1)
Student	127 (63.2)	119 (79.9)	0.001	212 (93.4)	174 (89.2)	0.13	632 (81.9)
Civil servant	4 (1.9)	1 (0.7)	0.31	3 (1.3)	2 (1.0)	0.78	10 (1.3)
No response	0 (0.0)	0 (0.0)	-	0 (0.0)	1 (0.5)	0.28	1 (0.1)
Total	201 (100.0)	149 (100.0)		227 (100.0)	195 (100.0)		772 (100.0)
Educational level							
Quaranic	0 (0.0)	1 (0.7)	0.42	0 (0.0)	0 (0.0)	-	1 (0.1)
Primary	33 (16.4)	10 (6.7)	0.006	7 (3.1)	21 (10.8)	0.002	71 (9.2)
Secondary	132 (65.7)	113 (75.8)	0.004	173 (76.2)	123 (63.1)	0.003	541 (70.1)
Tertiary	29 (14.4)	23 (15.5)	0.79	45 (19.8)	49 (25.1)	0.19	146 (18.9)
Others	7 (3.5)	2 (1.3)	0.31	2 (0.9)	2 (1.0)	1.000	13 (1.7)
Total	201 (100.0)	149 (100.0)		227 (100.0)	195 (100.0)		772 (100.0)
Years of residence in a location							
Less than 3 years	22 (10.9)	14 (9.4)		40 (17.6)	56 (30.3)		132 (17.1)
3 years or more	179 (89.1)	135 (90.6)	0.64	187 (82.4)	139 (69.7)	0.007	640 (82.9)
Total	201 (100.0)	149 (100.0)		227 (100.0)	195 (100.0)		772 (100.0)
Number of meals ate per days							
≥1 meal a day	13 (6.5)	4 (2.7)	0.05	3 (1.3)	4 (2.1)	0.71	23 (3.0)
2 meals a day	116 (57.7)	85 (57.0)	0.90	77 (34.0)	88 (45.1)	0.02	366 (47.4)
3 meals a day	72 (35.8)	60 (40.3)	0.49	146 (64.3)	101 (51.8)	0.009	379 (49.2)
No response	0 (0.0)	0 (0.0)	-	1 (0.4)	2 (1.0)	0.60	3 (0.4)
Total	201 (100.0)	149 (100.0)		227 (100.0)	195 (100.0)		772 (100.0)
Reported HIV status							
HIV negative	39 (19.4)	41 (27.5)	0.07	58 (25.6)	54 (27.7)	0.62	192 (24.9)
HIV untested/no response	162 (80.6)	108 (72.5)	0.07	167 (73.5)	140 (71.8)	0.18	577 (74.7)
HIV positive	0 (0.0)	0 (0.0)	-	2 (0.9)	1 (1.5)	1.000	3 (0.4)
Total	201 (100.0)	149 (100.0)		227 (100.0)	195 (100.0)		772 (100.0)
Intake of alcohol							
Yes	90 (44.8)	28 (18.8)		99 (43.6)	40 (20.5)		257 (33.3)
No	111 (55.2)	121 (81.2)	0.000	128 (56.4)	155 (79.5)	0.000	515 (66.7)
Total	201 (100.0)	149 (100.0)		227 (100.0)	195 (100.0)		772 (100.0)
Use of psychoactive substances							
None	194 (96.5)	149 (100.0)	0.02	214 (94.3)	195 (100.0)	0.001	752 (97.4)
One type	5 (2.5)	0 (0.0)	0.08	10 (4.4)	0 (0.0)	0.002	15 (1.9)
Two or more types	2 (1.0)	0 (0.0)	0.22	3 (1.3)	0 (0.0)	0.25	5 (0.6)
Total	201 (100.0)	149 (100.0)		227 (100.0)	195 (100.0)		772 (100.0)
Ever had sexual intercourse							
Yes	139 (69.2)	88 (59.1)		107 (47.1)	79 (40.5)		413 (53.5)
No	63 (30.8)	61 (40.6)	0.06	120 (52.9)	116 (59.5)	0.01	359 (46.5)
Total	201 (100.0)	149 (100.0)		227 (100.0)	195 (100.0)		772 (100.0)
Mean age (± SD) at sexual debut							
Mean age	17.2 ± 2.0	17.1 ± 2.1	1.00	18.3 ± 2.8	16.6 ± 2.5	0.000	15.1 ± 5.2
Ever had sexual intercourse							
Yes	139 (69.2)	88 (59.1)		107 (47.1)	79 (40.5)		413 (53.5)
No	63 (30.8)	61 (40.6)	0.06	120 (52.9)	116 (59.5)	0.01	359 (46.5)
Total	201 (100.0)	149 (100.0)		227 (100.0)	195 (100.0)		772 (100.0)
History of transactional sex							
Yes	2 (1.0)	10 (6.7)		7 (3.1)	9 (4.6)		28 (3.6)
No	199 (99.0)	139 (93.3)	0.005	220 (96.9)	186 (95.4)	0.41	744 (96.4)
Total	201 (100.0)	149 (100.0)		227 (100.0)	195 (100.0)		772 (100.0)
Awareness about condom							
Yes	195 (97.0)	141 (94.6)		226 (99.6)	194 (99.5)		756 (97.9)
No	6 (3.0)	8 (5.4)	0.17	1 (0.4)	1 (0.5)	1.000	16 (2.1)
Total	201 (100.0)	149 (100.0)		227 (100.0)	195 (100.0)		772 (100.0)
Knowledge of where to obtain condom							
Yes	201 (100.0)	149 (100.0)		220 (96.9)	180 (92.3)		750 (97.2)
No	0 (0.0)	0 (0.0)	-	7 (3.1)	15 (7.7)	0.03	22 (2.8)
Total	201 (100.0)	149 (100.0)		227 (100.0)	195 (100.0)		772 (100.0)
Ever used female condom							
Yes	12 (6.0)	6 (4.0)		4 (1.8)	3 (1.5)		25 (3.2)
No	189 (94.0)	143 (96.0)	0.42	223 (98.2)	192 (98.5)	0.86	747 (96.8)
Total	201 (100.0)	149 (100.0)		227 (100.0)	195 (100.0)		772 (100.0)
Knowledge of HIV transmission							
Good	121 (60.2)	79 (53.0)		162 (71.4)	119 (61.0)		481 (62.3)
Poor	80 (39.8)	70 (47.0)	0.18	65 (28.6)	76 (39.0)	0.03	291 (37.7)
Total	201 (100.0)	149 (100.0)		227 (100.0)	195 (100.0)		772 (100.0)
Knowledge of HIV prevention							
Good	110 (54.7)	76 (51.0)		104 (45.8)	106 (54.4)		396 (51.3)
Poor	91 (45.3)	73 (49.0)	0.49	123 (54.2)	89 (45.6)	0.08	376 (48.7)
Total	201 (100.0)	149 (100.0)		227 (100.0)	195 (100.0)		772 (100.0)

Significantly more female than male respondents in the urban area were in primary (16.4% vs 6.7%; p = 0.006) and secondary schools (65.7% vs 75.8%; p = 0.004) while there was no gender difference in the proportion of respondents in the tertiary institutions (14.4% vs 15.5%; p = 0.79). In the rural area, there were more female than male respondents in primary schools (3.1% vs 10.8%; p = 0.002), more male than female respondents in secondary schools (76.2% vs 63.1%; p = 0.003), and no significant difference in the proportion of male and female respondents in tertiary institutions (19.8% vs 25.1%; p = 0.19).

There was no significant gender difference in terms of access to three meals per day in the rural area (35.8% vs 40.3%; p = 0.49). However, in the urban area, significantly more males than females were able to access three meals per day (64.3% vs 51.8%; p = 0.009).

### Sexual Practices

The most common form of sexual practice was vaginal sex. 402 (97.3%) of sexually active respondents reported having vaginal sex, 36 (8.7%) had had oral sex and eight (1.9%) had had anal sex. Most of the respondents who reported anal sex (87.5%) resided in the urban area and were males (87.5%). In the last 12 months preceding the survey, anal sex was reported only during heterosexual relationships (Table 2).

**Table 2:** Sexual Profile of Sexually Active Respondents (n = 413).

Variables	Population						Total (%)
							N = 413
	Rural		P value	Urban		P value	
	N = 227			N = 186			
	Male (%)	Female (%)		Male (%)	Female (%)		
	n = 139	n = 88		n = 107	n = 79		
Reasons for sexual debut							
Love	76 (54.7)	64 (72.7)	0.006	53 (49.5)	44 (55.7)	0.41	237 (57.4)
Having fun	23 (16.6)	10 (11.4)	0.28	34 (31.8)	9 (11.4)	0.001	76 (18.4)
Peer pressure	38 (27.3)	12 (13.7)	0.02	14 (13.1)	4 (5.1)	0.08	68 (16.5)
For money	0 (0.0)	0 (0.0)	-	0 (0.0)	0 (0.0)	-	0 (0.0)
Forced	1 (0.7)	1 (1.1)	1.000	1 (0.9)	19 (24.1)	0.000	22 (5.3)
Other	1 (0.7)	1 (1.1)	1.000	5 (4.7)	3 (3.7)	1.000	10 (2.4)
Total	139 (100.0)	88 (100.0)		107 (100.0)	79 (100.0)		413 (100.0)
Sexually active in last 12 months							
Sexually active	95 (68.3)	53 (60.2)		82 (76.7)	64 (81.0)		294 (71.2)
Not sexually active	35 (25.2)	23 (26.1)	0.61	24 (22.4)	15 (19.0)	0.55	97 (23.5)
No response	9 (6.5)	12 (13.7)		1 (0.9)	0 (0.0)		22 (5.3)
Total	139 (100.0)	88 (100.0)		107 (100.0)	79 (100.0)		413 (100.0)
^+^Forms of sexual practices ever practiced							
Anal	1 (0.7)	0 (0.0)	1.000	6 (5.6)	1 (1.3)	0.12	8 (1.9)
Oral	12 (8.6)	8 (6.8)	0.91	10 (9.3)	6 (7.6)	0.67	36 (8.7)
Vagina	137 (98.6)	84 (95.5)	0.21	104 (97.2)	77 (97.5)	1.000	402 (97.3)
Form of sexual practice in the last 12 months							
Anal	0 (0.0)	0 (0.0)	-	4 (4.5)	0 (0.0)	0.15	4 (1.0)
Oral	8 (5.8)	6 (6.8)	0.13	6 (5.6)	2 (2.5)	0.29	22 (5.3)
Vagina	116 (83.5)	71 (80.7)	0.70	86 (97.7)	65 (82.3)	0.87	338 (81.8)
^+^ Forms of sexual practices with spouse in the last 12 months							
Anal	0 (0.0)	0 (0.0)	-	4 (4.5)	0 (0.0)	0.14	4 (1.0)
Oral	0 (0.0)	1 (1.1)	0.39	1 (0.9)	0 (0.0)	1.000	2 (0.5)
Vagina	5 (3.6)	18 (20.5)	0.000	8 (7.4)	12 (15.2)	0.09	43 (10.4)
^+^ Forms of sexual practices with boy/girlfriend in the last 12 months							
Anal	0 (0.0)	0 (0.0)	-	1 (0.9)	0 (0.0)	1.000	1 (0.3)
Oral	6 (4.3)	6 (6.8)	0.41	6 (5.6)	2 (2.5)	0.31	20 (6.8)
Vagina	90 (64.7)	45 (51.1)	0.04	77 (72.0)	61(77.2)	0.42	273 (92.9)
^+^ Forms of sexual practices with commercial sex worker in the last 12 months							
Anal	0 (0.0)	0 (0.0)	-	0 (0.0)	0 (0.0)	-	0 (0.0)
Oral	0 (0.0)	0 (0.0)	-	0 (0.0)	0 (0.0)	-	0 (0.0)
Vagina	0 (0.0)	0 (0.0)	-	7 (6.5)	0 (0.0)	0.02	7 (1.7)
^+^ Forms of sexual practices with casual sex partner in the last 12 months							
Anal	0 (0.0)	0 (0.0)	-	1 (0.9)	0 (0.0)	1.000	1 (0.2)
Oral	6 (4.3)	6 (6.8)	0.54	4 (4.5)	2 (2.5)	1.000	18 (4.4)
Vagina	2 (1.4)	2 (2.3)	0.64	12 (11.2)	11 (12.2)	0.56	27 (6.5)
Anal sex with man/woman in last 12 months							
Man	0 (0.0)	0 (0.0)	-	0 (0.0)	0 (0.0)	-	0 (0.0)
Woman	0 (0.0)	0 (0.0)	-	4 (4.5)	0 (0.0)	0.14	4 (1.0)
Number of current sexual partners							
0	36 (25.9)	24 (27.2)	0.82	23 (21.5)	14 (17.7)	0.52	97 (23.5)
1	46(33.1)	51 (58.0)	0.000	13 (12.1)	57 (72.2)	0.000	167 (40.4)
>1	56 (40.3)	10 (11.4)	0.000	30 (20.0)	8 (10.1)	0.003	104 (25.2)
No response	1 (0.7)	3 (3.4)	0.30	41 (38.4)	0 (0.0)	0.000	45 (9.9)
Total	139 (100.0)	88 (100.0)		107 (100.0)	79 (100.0)		413 (100.0)
^a^Use of condom at last sexual act							
Anal	1 (0.7)	0 (0.0)	1.000	3 (2.8)	0 (0.0)	0.26	4 (1.0)
Oral	1 (0.7)	1 (1.1)	1.000	2 (1.9)	0 (0.0)	0.51	4 (1.0)
Vagina	95 (68.3)	52 (59.1)	0.16	74 (69.1)	41 (51.9)	0.02	262 (63.4)
Reason for use of male condom							
Prevent HIV/STI	15 (10.8)	3 (3.4)	0.05	17 (15.9)	2 (2.5)	0.003	37 (9.0)
Prevent pregnancy	3 (2.2)	0 (0.0)	0.29	3 (2.8)	1 (1.3)	0.64	7 (1.7)
Prevent both	67 (48.2)	35 (39.8)	0.21	44 (41.1)	32 (40.5)	0.93	178 (43.1)
No response	12 (8.6)	15 (17.0)	0.06	15 (14.1)	6 (6.8)	0.17	48 (11.6)
^+^History of STI in last 12 months							
Discharge	5 (3.6)	9 (10.2)	0.04	5 (4.7)	21 (26.6)	0.000	40 (9.7)
Itching	2 (1.4)	15 (17.0)	*0.000	16 (15.0)	23 (29.1)	0.02	56 (13.6)
Sore	1 (0.7)	4 (4.5)	0.06	3 (2.8)	6 (7.6)	0.17	14 (3.4)

^a^ Multiple responses possible

Only male respondents reported sex with commercial sex workers in both urban and rural areas. Oral and anal sex was not reported with commercial sex workers.

More male respondents resident in the urban area reported having sex with casual partners (7.0% vs 15.3%; p = 0.007). See Table 2. Anal, oral and vaginal sex was reported with spouses, boy/girlfriends and casual sex partners. Anal sex was most often reported in spousal relationships.

### Sexual Behavior

Four hundred and thirteen (53.5%) respondents reported being sexually active. This include 139 (33.7%) of 15–19 year olds and 274 (66.3%) 20–24 year olds. More respondents in the rural than urban area reported being sexually active (64.9% vs 44.1%; p<0.001). See Table 1. More male than female respondents resident in the urban area reported been sexually active (47.1% vs 40.5%; p = 0.01) while no statistically significant difference in sexual activity was observed in proportion of male and female respondents in the rural area (69.2% vs 59.1%; p = 0.06).

There was no statistically significant difference in the mean age of sexual debut of male and female respondents resident in the rural area (17.2yrs vs 17.1yrs; p = 1.00), while female respondents in the urban area initiated sex at a significantly earlier age than males (18.3yrs vs 16.6 yrs; p<0.001). The mean age of sexual debut among females in the urban area was also significantly lower than the mean age of sexual debut for female in the rural area (16.6yrs vs 17.1yrs; p = 0.005).

Reasons given for sexual debut ranged from love (57.4%), to having fun (18.4%), peer pressure (16.5%) and forced sex initiation (5.3%). No respondents reported initiating sex for money.

In the rural area, the main reason for sexual debut for respondents was love, with significantly more female than male respondents reporting this (54.7% vs 72.7%; p = 0.006). The secondary reason for sexual debut was peer pressure, with more male than female than male respondents listing it as the reason for sexual debut (27.3% vs 13.7%; p = 0.02). There was no statistically significant difference in the proportion of male and female respondents who identified ‘having fun’ (16.6% vs 11.4%; p = 0.28) and ‘forced sex initiation’ (0.7% vs 1.1%; p = 1.00) as reasons for sexual debut.

In the urban area, the main the reason given for sexual debut for both male (49.5%) and female (55.7%) respondents was love. There was no statistically significant difference in the proportion of male and female respondents who identified love (49.5% vs 55.7%; p = 0.41) or peer pressure (13.1% vs 5.1%; p = 0.08) as reasons for sexual debut. However, more male than female respondents identified ‘having fun’ as the reason for sexual debut (31.8% vs 11.4%; p = 0.001) while more female than male respondents identified ‘forced sex initiation’ as the reason for sexual debut (0.9% vs 24.1%; p<0.001).

Transactional sex was practiced by both male and female respondents resident in urban and rural areas. Significantly more female than male respondents resident in the rural area gave a history of transactional sex (1.0% vs 6.7%; p = 0.005).

Over 90% of respondents reported knowing about condoms. Every respondent in the rural area knew where to obtain the condom, while significantly more male than female respondents in the urban area knew where to obtain condoms (96.9% vs 92.3%; p = 0.03). Very few respondents (3.2%) reported ever using the female condom.

There was no statistically significant difference in condom use at last vaginal sex act between respondents resident in rural and urban areas (65% vs. 62%; p = 0.54). However, significantly more male respondents in the urban area used condom during the last vaginal sexual intercourse (69.1% vs 51.9%; p = 0.02). The main reason for use of condom during sex was to prevent HIV infection and pregnancy.

Also, 104 (25.2%) respondents reported having more than one sexual partner, with significantly more respondents from the rural than urban area this (29.5% vs 20.4%, p = 0.04). In both rural (40.3% vs 11.4%; p<0.001) and urban (20.0% vs 10.1%; p = 0.003) areas, more male than female respondents reported having more than one current sex partner.

### HIV risk profile

There were very few respondents (0.4%) who reported being HIV positive, and all were from the urban area. Most of the respondents had good knowledge of HIV prevention (51.3%) and HIV transmission (62.3%). However, the proportion of respondents who could correctly identify modes of HIV transmission was significantly higher than those who could correctly identify ways off prevention HIV infection (62.3% vs 51.3%; p<0.001). While there were no gender differences in knowledge of HIV prevention of respondents of both rural (54.7% vs 51.0%; p = 0.49) or urban (45.8% vs 54.4%; p = 0.08) areas, more male than female respondents in the urban area had better understanding about HIV transmission (71.4% vs 61.0%; p = 0.03).

Gender differences were observed in the use of alcohol and psychoactive substances. More male than female respondents in both urban (43.6% vs 20.5%; p<0.001) and rural (44.8% vs 18.8%; p<0.001) areas drank alcohol. Only male respondents reported the use of psychoactive substances.

More female than male respondents in both urban and rural areas reported symptoms of STIs. More females than males in both rural (3.6% vs 10.2%; p = 0.04) and urban (4.7% vs 26.6%; p<0.001) areas gave a history of discharge. More females than males in both rural (1.4% vs 17.0%; p = 0.04) and urban (15.0% vs 29.1%; p<0.001) areas also gave a history of itching.

## Discussion

Our study highlights significant differences in the HIV sexual risk profile of adolescents and young persons’ resident in urban and rural Nigeria. Specifically, more respondents resident in the rural than urban area were sexually active and reported having more than one sexual partner; while more respondents in the urban than rural area reported anal sex. The HIV sexual risk profile differed significantly by gender and residential area, with more male than female respondents being sexually active, knowing where to obtain condoms using condom during the last vaginal sexual intercourse, and reporting sex with casual partners. On the other hand, the age of sexual debut for female respondents was lower than that for male respondents. In the rural area, more female than male respondents engaged in transactional sex. In both urban and rural areas, more male than female respondents had multiple sexual partners, and more females than males reported symptoms of STI.

Urban-rural residence is an important variable that influences reproductive health outcomes. The distinction between HIV sexual risk profile of adolescents and young persons’ resident in urban and rural Nigeria is important because of differences in access to health care services, cultural beliefs, and living situations in these two areas [19]. The observed differences in sexual behavior, sexual practices and HIV risk profile of urban and rural dwelling adolescents and young persons’ suggest that adolescent and youth socialization process in these residential areas may inform their sexual behavior, sexual practices and sexual health choices. Studies [20,21] have demonstrated the impact of the mass media on the context of adolescents’ sexual behavior. The difference in access and exposure of adolescents and young persons’ resident in rural and urban areas to the sexually explicit media may account for the differences observed in sexual practices. This however, cannot totally explain the observed gender differences in sexual behaviors reported in this study.

We note that a conservative number of respondents reported anal sex, and anal sex was reported only in heterosexual spousal relationships 12 month preceding the survey. This is in contrast to reports by adolescents 15–19years in a prior survey reported from Nigeria [6]. This report may reflect a reporting bias as anal sex is stigmatized [22]. It may also reflect the conservative nature of the study population as the study was conducted in urban areas which are less cosmopolitan than many of the urban areas where Folayan et al [6] conducted their study.

Our findings of the study suggests the need to design different HIV prevention programs for adolescents and young persons’ resident in urban and rural settings in Nigeria bearing in mind difference in sexual behavior and practices. Gender differences in programs within urban and rural areas also need to be considered. Gender differences in HIV risk behavior in residential areas had also been earlier identified. Voeten et al [23] identified that more women in rural than urban areas in Tanzania engaged in risky sexual behavior. This study, unlike the report of Voeten et al [23], showed that the behavior of adolescents and young women in rural and urban areas were not homogenous: there are gender differences observed in the HIV risk profile irrespective of the residential area.

The large proportion of sexually active female adolescents and young persons’ resident in urban areas who reported forced sex initiation is a cause for concern. Folayan et al [24] had highlighted the need to improve public dialogue on the prevention of rape and to explore the possible link between HIV infection and rape in Nigeria [6]. There has been no study examining the causal relationship between rape and HIV in Nigeria, pointing to a need [6,24]. Where studies provide evidence for such link, rape can then be recognized as a HIV risk factor in Nigeria, and appropriate programs can be designed to prevent and mitigate its impact.

Our study has its limitations. First, while we have a large sample size, it is not generalizable to all of Nigeria. Second, the selection of participants who were more likely to openly discuss sex inherently introduced a bias in the study sample. Third, the use of an interviewer administered questionnaire to ask sensitive questions on sex from adolescents and young persons is fraught with the prospect for under-reporting of history of sexual intercourse. Finally, self-reporting of HIV status limits the dependability of the true HIV test result. Despite these limitations, the rigorous methodology employed to generate the study can be used to design a pilot HIV and STI sexual prevention programs for adolescents and young persons’ in Nigeria.

## Conclusion

We showed significant differences in the sexual practices, sexual behavior, and HIV risk profile of adolescents and young adults resident in urban and rural Nigeria. Our study provides evidence to support the need for differential HIV and STI prevention programming by age and gender in Nigeria. Sexual and reproductive health education programs for adolescents and young adults in Nigeria need to include information about safe sexual practices including prevention and management of STIs. Finally, the additional evidence on the high proportion of females whose sexual debut is through forced sex initiation reinforces the need to conduct studies on the prevalence of rape and causal relationship between HIV and rape so that appropriate programming for rape prevention and management can be instituted.
